# HSV‐1 US3: A Master Mimic Hijacking Host Kinase Networks for Viral Dominance

**DOI:** 10.1002/jmv.70735

**Published:** 2025-12-04

**Authors:** Imran Hossain, Christella J. Nelson, Ojasvi Dutta, Harikrishnan Mohan, Mehedi Hasan, Konstantin G. Kousoulas

**Affiliations:** ^1^ Department of Pathobiological Sciences Louisiana State University School of Veterinary Medicine Baton Rouge Louisiana USA; ^2^ Division of Biotechnology and Molecular Medicine School of Veterinary Medicine Baton Rouge Louisiana USA

**Keywords:** antiviral targets, Herpes Simplex Virus 1, kinase signaling, molecular mimicry, US3 kinase

## Abstract

Herpes simplex virus type 1 (HSV‐1) encodes the serine/threonine kinase US3, a central regulator of host–virus interactions that promotes viral replication, latency, and reactivation. Despite limited sequence homology to cellular kinases such as Akt or PKA, US3 phosphorylates diverse substrates to manipulate multiple pathways. This review summarizes current knowledge of US3 functions across several major areas, including suppression of innate immune responses through modulation of NF‐κB, ERK/MAPK, TBK1/IRF3, RIG‐I; promotion of cell survival via phosphorylation of canonical Akt substrates such as TSC2, FOXO1/3, and BAD; and facilitation of nuclear egress through modification of lamin A/C, emerin, and the nuclear egress complex proteins UL31 and UL34. By comparing US3's strategies with those of cellular kinases and other viral effectors, this review highlights convergent mechanisms of host manipulation. A comprehensive understanding of US3's multifunctional activities advances insight into HSV‐1 pathogenesis and underscores its potential as a target for novel antiviral therapies.

## 1 Introduction

Protein kinases regulate cellular processes by catalyzing phosphorylation of serine, threonine, or tyrosine residues, thereby altering protein activity, localization, and interactions. These enzymes activate the transfer of the gamma phosphate from ATP to specific serine, threonine, or tyrosine residues on their target proteins, thereby regulating fundamental processes such as signal transduction, cell cycle progression, metabolism, and immune responses [1]. Consequently, kinase signaling pathways represent frequent and critical targets for pathogens aiming to commandeer host cell resources and skillfully evade defensive measures [2].

Herpes simplex virus type 1 (HSV‐1), a neurotropic alpha herpesvirus, is responsible for a variety of human illnesses, including mild conditions like cold sores and severe ones like encephalitis (brain inflammation) [3]. HSV‐1 encodes several proteins that manipulate host functions, including its two known protein kinases, UL13 and US3. UL13 is a conserved herpesvirus protein kinase (CHPK) that regulates viral gene expression, virion maturation, and immune modulation. In contrast, US3 is uniquely present in alpha herpesviruses and absent in beta‐ and gamma herpesviruses. This viral kinase exhibits broad functional diversity and manipulates host kinase networks in ways that resemble cellular kinases [4, 5].

US3, a serine/threonine kinase encoded within the unique short (US) region of the HSV‐1 genome that has several distinct functions. It regulates nuclear egress, actively prevents host cell apoptosis, modulates the cytoskeleton, and suppresses innate and adaptive immune responses [6]. US3 hijacks host kinase pathways through the phosphorylation of key host and viral substrates, emulating the action of cellular kinases like Akt or PKA, despite a conspicuous lack of significant primary sequence homology [7].

This review presents the diverse functions of HSV‐1 US3. We delve into the structural underpinnings of its functionality and explore the specific molecular mechanisms by which US3 manipulates or mimics host kinase signaling pathways integral to immune evasion, cell survival, and nuclear egress. US3 directly and/or indirectly modifies numerous host and viral protein discussed in the following sections and summarized in Table 1. By drawing comparisons between US3's strategies and those of its cellular counterparts and other viral effectors, we aim to provide a novel perspective on how this viral kinase modulates cellular pathways for viral replication, thereby highlighting its significant potential as a therapeutic target.

**Table 1:** Known substrates of HSV‐1 US3 kinase, their phosphorylation context, and functional consequences.

Target	Host/Viral	Species/Context	Evidence	Site/Notes	Functional consequence	References
RIG‐I (DDX58)	Host	HSV‐1	Direct phosphorylation	Ser‐8	Blocks TRIM25‐mediated ubiquitination; reduces RIG‐I‐MAVS binding; suppresses type I IFN induction	[8]
IRF3	Host	HSV‐1	Direct phosphorylation	Ser‐175	Blocks IRF3 activation (dimerization & nuclear translocation); inhibits IFN‐ß production	[9]
RelA/p65	Host	HSV‐1	Direct phosphorylation	Ser‐75	Blocks nuclear translocation; dampens NF‐κB signaling	[10]
m6A methyltransferase complex (METTL3/METTL14/WTAP)	Host	HSV‐1 (also conserved in PRV)	US3‐dependent phosphorylation and inactivation	Hyperphosphorylation observed; no sites identified yet	Loss of m6A modification on host mRNA impairs RNA metabolism, weakens innate immune responses	[11]
Lamin A/C (LMNA/C)	Host	HSV‐1	Direct phosphorylation	Multiple sites ‐ hyperphosphorylation observed, but exact Ser/Thr residues phosphorylated by US3 remain unmapped in HSV‐1	Disassembly of nuclear lamina; facilitates nuclear egress	[12, 13, 14]
Emerin (EMD)	Host	HSV‐1	US3‐dependent phosphorylation/relocation	Hyperphosphorylation and redistribution (specific amino acid residues not mapped)	Nuclear envelope remodeling for egress	[12, 13, 14]
Matrin‐3 (MATR3)	Host	HSV‐1/PRV/VZV homologs	phosphorylation is US3/ORF66 dependent, and VZV ORF66 mutation of T150 reduces phosphorylation, but direct in vitro phosphorylation by US3 in purified system has not been conclusively shown	T150 is novel in VZV; US3 dependence shown in HSV‐1/PRV; mutation confirms the importance of T150.	Alters nucleo‐cytoplasmic transport; affects nuclear environment	[15]
HDAC1/HDAC2	Host	HSV‐1 (observed in VZV US3 ortholog ORF66)	US3‐dependent phosphorylation	Hyperphosphorylation, but specific amino acid sites not mapped	Relieves chromatin repression; promotes viral transcription	[15, 16, 17]
TSC2	Host	HSV‐1	Direct phosphorylation (Akt‐mimicry)	Ser939/Thr1462 (Akt sites)	Constitutive mTORC1 activation; supports replication	[18]
FOXO1/FOXO3	Host	HSV‐1	US3 drives phosphorylation on Akt sites (direct or Akt‐mimic)	Thr24/Ser256/Ser319	Nuclear exclusion of FOXO transcription factors; suppression of apoptosis	[18]
Bad	Host	HSV‐1	Direct phosphorylation	Not specified	Antiapoptotic; prevents Bad binding Bcl‐2/Bcl‐XL	[19]
Bid	Host	HSV‐1	Direct phosphorylation	Not specified	Prevents granzyme B‐mediated processing; blocks apoptosis	[20]
Procaspase 3	Host	HSV‐1	Direct phosphorylation	Not specified	Blocks caspase‐3 activation; antiapoptotic	[19]
?‐catenin (CTNNB1)	Host	HSV‐1	Direct phosphorylation	Thr‐556	Blocks nuclear entry; suppresses cGAS/STING pathway	[9, 21]
KIF3A	Host	HSV‐1	Direct phosphorylation	Primary site is Ser‐687, possible second site is Thr‐633	Disrupts recycling of membrane proteins; facilitates immune evasion	[22]
Group A p21‐activated kinases (PAKs)	Host	HSV‐1/PRV	US3 binds to and phosphorylates	Not specified	Leads to actin cytoskeleton rearrangements; promotes intercellular viral spread	[23]
Cofilin pathway	Host	PRV/HSV1	US3 modulates cofilin phosphorylation via PAK/LIMK	Not specified	Actin remodeling; facilitates viral entry and cell‐to‐cell spread	[24, 25]
PKA pathway	Host	HSV‐1	US3 activates pathway	Not specified	Leads to phosphorylation of PKA substrates; antiapoptotic functions	[26]
MHC‐I	Host	HSV‐1	Indirectly required for downregulation	Not specified	Aids in evasion of CD8 + T‐cells	[27]
CD1d	Host	HSV‐1	US3 impairs recycling via KIF3A phosphorylation	Primary site is Ser‐687, Possible second site is Thr‐633	Evades detection by NKT cells	[22]
ERK1/2 (MAPK)	Host	HSV‐1	US3 indirectly suppresses activity	Not specified	Promotes cell survival	[28]
Cytokeratin 17	Host	HSV‐2	Direct phosphorylation by US3	Not specified	F‐actin disassembly, immune evasion, apoptosis inhibition	[29]
GBP1	Host	PRV	US3 phosphorylates	Not specified	Not specified	[30, 31]
Rho GTPases	Host	PRV	US3 can phosphorylate	Not specified	Changes in cytoskeleton	[23, 32]
Bclaf1	Host	HSV1/PRV	Degradation by US3 (proteasome‐dependent)	Not specified	Suppresses IFN‐α–induced STAT1/2 phosphorylation and ISG transcription; dampens antiviral state	[33]
MHC class I‐related gene protein (MR1)	Host	HSV‐1	Downregulated by US3	Not specified	Impairs MAIT cell antigen presentation; evasion of innate‐like T cell immunity	[34]
UL31	Viral	HSV‐1	Direct phosphorylation by US3	Multiple serines	Proper NEC localization; primary envelopment	[12]
UL34	Viral	HSV‐1	Direct phosphorylation by US3	Thr‐195 and Ser‐198	Regulates NEC function; lamina disruption	[35]
UL47/VP11/12	Viral	DPV, HSV‐1	US3‐dependent phosphorylation	DPV: Thr29, Ser30, Ser42, Thr47, Ser161, and Thr775	Alters subcellular localization; promotes packaging into virions	[36]
gB (UL27)	Viral	HSV‐1	Direct phosphorylation by US3	Thr‐887 in cytoplasmic tail	Downregulates surface expression; impacts egress and cell‐cell spread	[37, 38]
dUTPase (UL50)	Viral	HSV‐1	Direct phosphorylation by US3	Ser‐187	Regulates catalytic activity; dictates CNS pathogenicity	[39]
ICP22	Viral	HSV‐1	Direct phosphorylation by US3 in vitro	Not specified	Not specified	[35]
Us9	Viral	HSV‐1	Direct phosphorylation by US3	Not specified	Functional consequence under investigation	[35]
US8A	Viral	HSV‐1	US3‐dependent phosphorylation	Ser‐61	Linked to neurovirulence and yields in brain	[40]
US3	Viral	HSV‐1	Autophosphorylation	Ser‐147	Regulates its own activity and localization	[41]

### 1.1 HSV‐1 US3: A Multifunctional Kinase

The concept of “moonlighting” proteins aptly describes single polypeptides that perform multiple, often seemingly unrelated, biological functions [42]. HSV‐1 US3 is a prime example of this phenomenon, functioning as a serine/threonine kinase while orchestrating a diverse array of processes critical for viral success. Its core kinase activity enables phosphorylation of serine or threonine residues on a wide range of both viral (e.g., UL31, UL34, gB, ICP22) [35, 37, 43] and cellular (e.g., p65, ERK1/2, RIG‐I, FOXO1, TSC2, BAD, lamin A/C, β‐catenin, Emerin, HDAC1/2) [6, 8, 10, 16, 18, 21, 28, 43, 44, 45, 46] targets.

### 1.2 Structural Basis for US3 Functional Diversity

**Figure 1:**
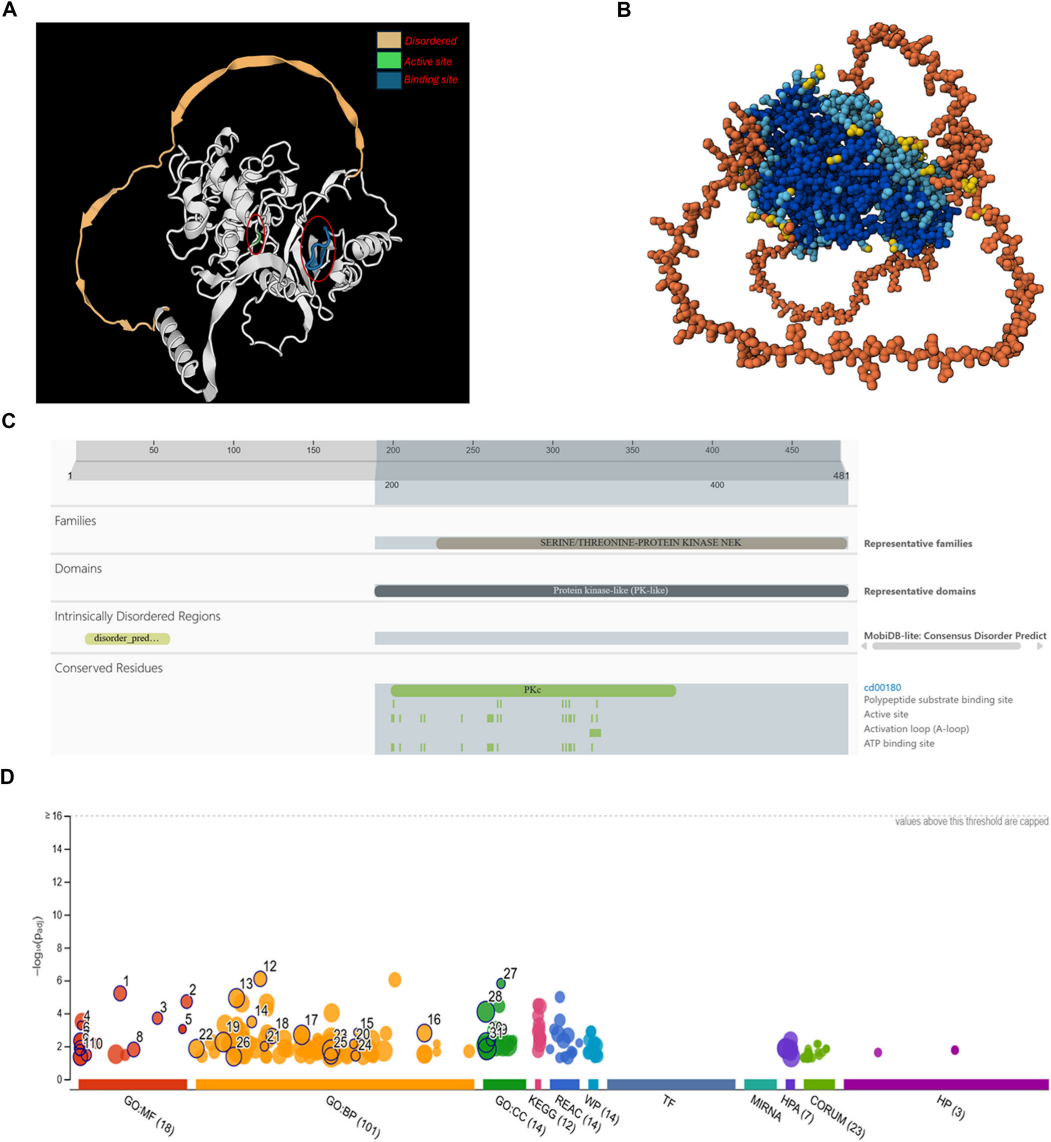
Structural prediction and functional enrichment analysis of HSV‐1 US3 kinase. (a) SWISS‐MODEL homology model of HSV‐1 US3 (downloaded from UniProt and manually annotated). The conserved protein kinase domain (residues 191–478) is shown in white, while a potentially disordered N‐terminal segment (residues 12–63) is depicted in light beige. The active site includes the catalytic base Asp305 and key residues of the ATP‐binding pocket (residues 197–205 and 220). This is a predicted structure based on sequence homology; the actual structure may differ, particularly in the non‐conserved regions. (b) AlphaFold3 (2024 release)‐predicted structure of US3, shown as a pLDDT confidence map in space‐fill representation. High‐confidence residues (pLDDT > 90) within the kinase core are colored dark blue, moderate‐confidence regions (70–90) in light blue, and low‐confidence/disordered regions ( < 70) in yellow–orange, corresponding mainly to the flexible termini. (This is a predicted structure based on sequence homology; the actual structure may differ, particularly in the non‐conserved regions). (c) Domain and motif annotation of US3 from InterPro/UniProt), showing its classification within the serine/threonine‐protein kinase family, conserved catalytic motifs, and predicted intrinsically disordered regions. (d) Functional enrichment of known and predicted US3 substrates analyzed using g:Profiler. The bubble plot displays significantly enriched functional categories across major databases. X‐axis: Functional annotation groups, including gene ontology—molecular function (GO:MF), biological process (GO:BP), and cellular component (GO:CC)—as well as reactome (REAC), KEGG pathways, CORUM complexes, transcription factors (TF), and protein–RNA interactions (MiRNA, HP). Y‐axis: Significance of enrichment expressed as −log₁₀(*p*‐value). Bubble size: Number of US3 substrates annotated within each term. Bubble color: Database source category. Numbers inside bubbles: Rank order of enrichment significance. Enriched terms cluster into three dominant categories—chromatin regulation, immune signaling, and RNA modification—supporting the broad functional spectrum of US3. (In addition to predictive models, recent structural and biochemical work (PMID: 40586577) demonstrates that US3 activity is dynamically regulated by autophosphorylation and phosphorylation by UL13, which enhance catalytic efficiency and may reconfigure substrate binding. This supports the notion that flexible, phosphorylation‐dependent structural tuning underlies US3's broad functionality [47].

Although a high‐resolution structure of full‐length US3 remains elusive, predictive modeling provides important insights into its architecture and functional potential (Figure 1). A homology‐based SWISS‐MODEL prediction (Figure 1a) highlights the canonical protein kinase domain (residues ~ 191–478 in HSV‐1, although numbering can vary slightly between strains). This region contains the catalytic base Asp305 and an ATP‐binding pocket located between residues ~ 197–205 and 220, both of which are essential for kinase activity. Notably, the N‐terminal extension ( ~ 12–63 residues) is predicted to be intrinsically disordered, while the C‐terminal tail also shows evidence of disorder. Such flexible segments likely provide regulatory interfaces for substrate docking or context‐specific interactions, a feature consistent with viral kinases that must act on diverse host targets [48].

AlphaFold3 (2024 release) predictions further support this model (Figure 1b) [49], where high‐confidence regions (pLDDT > 90, shown in the dark blue) correspond to the conserved kinase fold, while the termini are predicted with lower confidence (yellow–orange), reflecting structural flexibility or intrinsic disorder. These disordered regions may enable US3 to interact with multiple host proteins in pathways spanning from actin remodeling to immune signaling. Bioinformatic domain annotation from UniProt/InterPro (Figure 1c) confirms US3's classification as a serine/threonine kinase with conserved catalytic motifs, including the ATP‐binding loop, activation loop, and proton acceptor sites [50]. Importantly, the structured kinase domain is flanked by disordered extensions, reinforcing the idea that US3 has retained a stable enzymatic core while evolving flexible segments that expand substrate scope compared to canonical cellular kinases such as Akt or PKA.

Functional enrichment analysis of experimentally validated and putative US3 substrates using g:Profiler (Figure 1d) identified several significantly enriched biological themes [51]. The x‐axis represents different functional annotation categories, including gene ontology—molecular function (GO:MF), biological process (GO: BP), and cellular component (GO:CC)—as well as Reactome, KEGG, and CORUM databases. The y‐axis indicates the statistical significance of enrichment as −log₁₀(*p*‐value), with higher bubbles reflecting stronger enrichment. Each bubble corresponds to an enriched term, and the numbers within bubbles reference the ranked order of enrichment significance. Bubble color denotes the database source, while bubble size reflects the number of genes associated with that term. Collectively, these enrichments cluster into three major functional modules: chromatin regulation (e.g., DNA binding, histone deacetylase activity, NuRD complex), immune signaling (e.g., NF‐κB binding, apoptotic processes, cytokine regulation), and RNA modification (e.g., m⁶A methyltransferase activity, mRNA binding, RNA processing). These clusters are consistent with established US3 activities, including phosphorylation of HDAC1/2 to relieve chromatin repression, modulation of NF‐κB, IRF3, and RIG‐I to suppress innate immunity, and interference with the m⁶A methyltransferase complex to regulate RNA stability.

Recent structural and biochemical analyzes (PMID: 40586577) further refine our understanding of US3 regulation and substrate selection. Beyond predictive modeling, experimental evidence shows that US3 activity is modulated by both autophosphorylation and phosphorylation by the viral CHPK UL13, particularly at Ser147. These modifications enhance catalytic efficiency and may reconfigure the substrate‐binding pocket to accommodate distinct host and viral proteins. Importantly, the conformational flexibility provided by intrinsically disordered N‐ and C‐terminal segments enables US3 to engage a broad spectrum of substrates across immune, apoptotic, and nuclear egress pathways [7]. Together, these findings support a model in which phosphorylation‐dependent structural tuning, coupled with disordered regions, underlies US3's broad range of functions.

In summary, the structural landscape of US3 reveals a conserved kinase core that ensures catalytic activity, flanked by disordered terminal regions that provide flexibility for diverse interactions. This combination, further fine‐tuned by autophosphorylation and UL13‐mediated regulation, explains how US3 may achieve broad substrate specificity and context‐dependent functionality. This structural architecture allows US3 to function not simply as an Akt‐like mimic but as a versatile kinase that modulates its activity across distinct stages of the HSV‐1 lifecycle.

### 1.3 US3 as a Master Modulator of Cellular Signaling Pathways

US3's strategy centers on manipulating host signaling pathways. It achieves this by phosphorylating key host and viral proteins, sometimes functionally substituting for host kinases and sometimes directly interfering with pathway components to achieve a desired outcome (subversion). This interference enables HSV‐1 to hijack key pathways governing immunity, cell survival, and viral transport or release often bypassing normal cellular checkpoints [48]. The subsequent sections provide detailed, illustrative examples of US3's strategies in action.

### 1.4 Modulation of Immune Evasion Pathways

**Figure 2:**
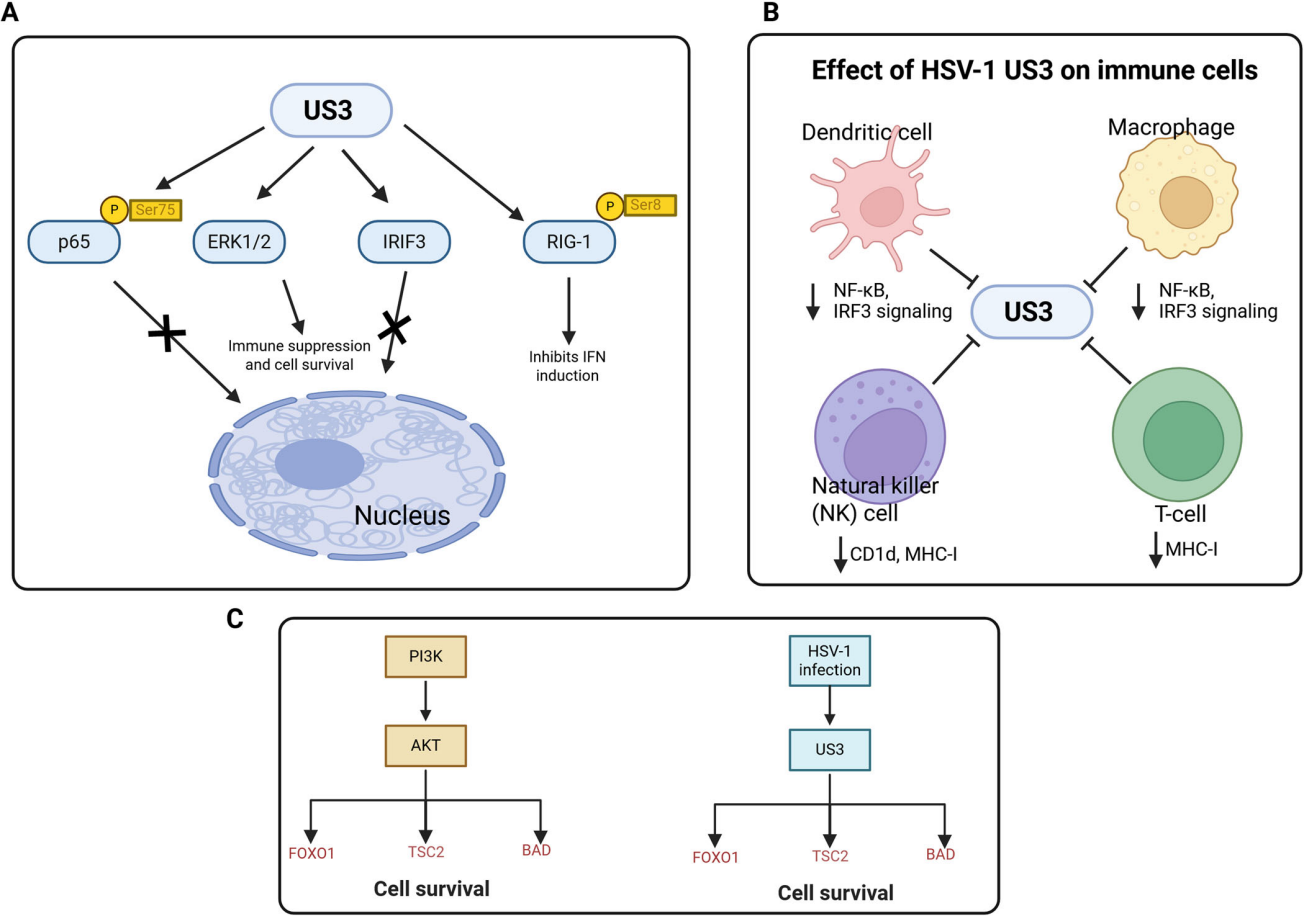
HSV‐1 US3 suppresses host antiviral signaling, modulates immune cell function, and promotes cell survival through Akt‐like kinase activity. (a) US3 directly or indirectly targets key immune regulators—p65 (pSer75), IRF3, and RIG‐I (pSer8), and modulates ERK1/2—to block NF‐κB and IFN‐I responses and promote cell survival. These actions collectively dampen antiviral defenses and support viral persistence. (b) Effect of HSV‐1 US3 on immune cells. US3 suppresses dendritic cell function by inhibiting NF‐κB and IRF3 signaling, leading to reduced cytokine and interferon production. In macrophages, US3 similarly dampens NF‐κB/IRF3 pathways, limiting inflammatory responses. US3 downregulates CD1d and MHC‐I, impairing NK/NKT cell recognition and T cell antigen presentation, thereby weakening both innate and adaptive antiviral immunity. (c) HSV‐1 US3 acts as a Akt‐like activity by phosphorylating key downstream targets—FOXO1, TSC2, and BAD—leading to mTORC1 activation and inhibition of apoptosis. These modifications create a pro‐survival environment that supports efficient viral replication.

Successful viral persistence relies on the virus's ability to circumvent host innate and adaptive immunity. HSV‐1 US3 targets multiple host proteins and pathways to suppress antiviral responses (Figure 2a).

### 1.5 Hijacking NF‐κB Signaling

The NF‐κB pathway is a central regulator of inflammation and antiviral gene expression [52]. Typically, stimuli such as TNF‐α trigger the activation of IKK, which leads to IκB degradation and the subsequent nuclear translocation of NF‐κB (p65/p50 dimer) to initiate transcription [53]. HSV‐1 US3 disrupts this signaling axis through its kinase activity, which is essential for blocking TNF‐α‐ and IL‐1β‐induced NF‐κB activation. US3 hyperphosphorylates the p65/RelA subunit at Serine 75, a modification that appears to impair p65 nuclear import and DNA binding, thereby suppressing the transcription of proinflammatory cytokines and interferon‐stimulated genes (ISGs). This action contrasts with canonical phosphorylation events (e.g., at Ser536) that typically enhance p65's transcriptional activity, representing an alternative mechanism of host pathway subversion [10]. Additionally, US3 may interfere with TRAF6 ubiquitination—an upstream event required for NF‐κB signaling activation [54]. While this strategy differs from that of other HSV‐1 immune evasion proteins such as ICP47 (which blocks TAP‐mediated peptide transport in human cells) [55], it shares the overall goal of silencing antiviral gene expression. Similar strategies are used by other herpesviruses, such as Epstein–Barr virus (EBV), whose BGLF4 kinase targets UXT to manipulate NF‐κB signaling [56].

### 1.6 Modulation of the ERK/MAPK Pathway

The ERK/MAPK pathway controls various cellular functions, including immune responses and apoptosis. Canonical activation involves MEK‐mediated phosphorylation of ERK1/2, initiating downstream signaling that can include antiviral gene expression and proapoptotic signals [57, 58]. US3 subverts this pathway by inducing sustained ERK1/2 phosphorylation. While the precise residues targeted by US3 or its downstream effects on ERK remain to be definitively mapped, this altered activation pattern appears to redirect signaling away from antiviral defenses and toward pro‐survival pathways. This results in reduced interferon‐induced apoptosis and prolongs infected cell viability, greatly benefiting viral replication and yield [28]. The sustained activation observed for ERK1/2 by US3 is atypical for many cellular stimuli, suggesting a direct or indirect persistent modification of the pathway.

### 1.7 Inhibition of the TBK1‐IRF3 Signaling Pathway

The TBK1–IRF3 signaling axis is crucial for type I interferon (IFN‐I) production following viral detection. Upon activation (e.g., by PRRs like RIG‐I or STING), TBK1 dimerizes and phosphorylates IRF3, enabling its dimerization, nuclear translocation, and binding to the IFN‐β promoter to activate transcription [59, 60]. US3 antagonizes this pathway in multiple ways. It directly phosphorylates IRF3, potentially inhibiting its activation, dimerization, or nuclear import [9]. Additionally, US3 has been shown to collaborate with the viral tegument protein UL46 to inhibit TBK1. UL46 disrupts TBK1 dimerization and activation, and this effect can be enhanced or modulated by US3 kinase activity [61]. These converging strategies effectively shut down IFN‐I induction. Notably, this immune evasion mechanism is also exploited by other herpesvirus kinases, such as the conserved CHPK (UL13) homologs, which similarly target IRF3 [62]. Recent work also suggests US3 can inhibit the STING pathway, upstream of TBK1, partially via hyperphosphorylation of β‐catenin, preventing its required nuclear translocation for IFN‐I production [21].

### 1.8 Phosphorylation of the RNA Sensor RIG‐I

Retinoic acid‐inducible gene I (RIG‐I) is a cytosolic pattern recognition receptor that detects viral RNAs and activates MAVS‐dependent signaling to induce IFN‐I. While RIG‐I is primarily associated with RNA virus sensing, it can also recognize altered host RNAs or viral RNAs produced during HSV‐1 infection [63]. Recent findings reveal that HSV‐1 US3 directly interacts with and phosphorylates RIG‐I at Serine 8 (S8) [8, 64]. This phosphorylation mimics a known negative regulatory host modification—normally carried out by cellular kinases—that inhibits RIG‐I's interaction with the E3 ubiquitin ligase TRIM25 [65, 66, 67, 68]. As a result, US3‐mediated phosphorylation at S8 blocks TRIM25 binding, prevents crucial RIG‐I ubiquitination required for its activation, and disrupts downstream MAVS recruitment, thereby suppressing IFN‐I signaling [8]. Consistent with this, a kinase‐dead HSV‐1 US3 mutant (K220A) fails to phosphorylate RIG‐I at S8 and elicits a significantly stronger IFN/ISG response in a RIG‐I‐dependent manner [8, 69]. This strategy appears conserved among alphaherpesvirus US3 homologs, reflecting a refined viral exploitation of host negative feedback systems governing PRR activation.

### 1.9 Downregulation of MHC Class I

US3 also contributes to evasion of adaptive immunity by downregulating surface expression of MHC class I molecules, impairing CD8 + T cell recognition of infected cells. Although US3 does not directly phosphorylate MHC‐I molecules, its kinase activity is essential for this effect [27]. The exact mechanism remains unclear but is distinct from ICP47‐mediated TAP inhibition or the general translational shutoff induced by the vhs protein [70]. Possible indirect mechanisms could involve modulation of endosomal trafficking, protein degradation pathways, or transcription factors regulating MHC‐I expression, all of which can be influenced by kinase activity. In murine models, inactivation of US3 kinase activity results in increased surface MHC‐I expression and significantly stronger HSV‐1‐specific CD8 + T cell responses and viral clearance. Conversely, depletion of CD8 + T cells enhances replication of US3 kinase‐dead virus in vivo, underscoring the functional significance of this immune evasion mechanism in physiological settings [27].

Beyond targeting intracellular signaling molecules, HSV‐1 infection broadly suppresses immune effector pathways at the cellular level. For example, HSV can impair dendritic cell maturation and antigen presentation, diminish NK cell recognition, and alter T cell responses, thereby weakening both innate and adaptive immunity. Recent studies also highlight the importance of non‐neutralizing antibody functions, such as antibody‐dependent cellular cytotoxicity (ADCC) and complement‐dependent cytotoxicity (CDC), in controlling HSV infection. Notably, HSV glycoproteins can actively interfere with ADCC responses, limiting Fc receptor‐mediated clearance of infected cells. These findings emphasize that HSV immune evasion extends well beyond kinase‐mediated signaling interference, encompassing systemic suppression of host immune pathways [71]. Figure 2b schematically summarizes US3‐specific interactions with dendritic cells, macrophages, NK/NKT cells, and T cells, highlighting how this kinase directly dampens immune cell functions to complement the broader viral immune evasion repertoire.

In summary, US3 employs multiple strategies to evade host immunity, including phosphorylation of NF‐κB p65, modulation of ERK/MAPK signaling, inhibition of TBK1–IRF3 activation, direct targeting of RIG‐I, and downregulation of MHC class I. Similar functions have been reported for the VZV US3 homolog ORF66, which downregulates CD1d and contributes to immune evasion, paralleling HSV‐1 US3 activities. These immune evasion substrates and their consequences are consolidated in Table 1.

### 1.10 Modulation of Pro‐Survival Signaling: US3 as an Akt Surrogate

To create an intracellular environment favorable for prolonged viral replication and particle production, viruses often evolve mechanisms to suppress host apoptotic pathways. The PI3K/Akt signaling cascade is a central regulator of cell survival, growth, and metabolism—making it a key target for viral manipulation [72]. HSV‐1 US3 plays a pivotal antiapoptotic role by exerting Akt‐like activity (Protein Kinase B) (Figure 2c).

### 1.11 Bypassing Akt to Activate mTORC1

The mechanistic target of rapamycin complex 1 (mTORC1) is a critical regulator of protein synthesis, cell growth, and autophagy. Its activity is normally restrained by the tuberous sclerosis complex (TSC), composed of TSC1 and TSC2 [73, 74]. Akt promotes mTORC1 activation by phosphorylating TSC2 at specific sites (e.g., Ser939 and Thr1462), thereby inhibiting TSC2's GTPase‐activating protein (GAP) activity toward the small GTPase Rheb [75, 76]. As a result, active Rheb‐GTP accumulates and activates mTORC1. Remarkably, US3 can also function by directly phosphorylating TSC2 at sites critical for its inactivation, including Ser939 and Thr1462 [18]. This US3‐mediated phosphorylation enables constitutive mTORC1 activation in infected cells—even under conditions where cellular Akt signaling is suppressed or when mTORC1 would typically be downregulated by stress sensors like AMPK or autophagy. Experimental studies show that depletion of TSC2 can rescue replication defects observed in US3‐deficient viruses, highlighting the importance of US3‐mediated TSC2 modification and subsequent mTORC1 activation for viral success [18]. Sustained mTORC1 activity facilitates phosphorylation and inactivation of 4E‐BP1, a translational repressor, thereby promoting cap‐dependent translation of viral mRNAs and suppressing host protein synthesis [18]. Other herpesviruses also manipulate this axis: Kaposi's sarcoma‐associated herpesvirus (KSHV) activates mTORC1 via proteins such as K1, vGPCR, or the viral kinase ORF36, while cytomegalovirus (CMV) utilizes UL38 to inhibit TSC2. However, US3's phosphorylation of TSC2 at canonical Akt‐regulatory sites represents a distinct viral strategy that converges on host signaling pathways [77].

### 1.12 Phosphorylation of Other Akt Substrates

US3's Akt‐like activity extends beyond TSC2. It also targets additional key host proteins involved in regulating apoptosis and metabolism that are canonical Akt substrates. For example, US3 phosphorylates the proapoptotic transcription factor FOXO1 at the same sites phosphorylated by Akt (Thr24, Ser256, Ser319 in human FOXO1) [28, 78]. This modification leads to FOXO1 inactivation and nuclear exclusion, thereby suppressing the transcription of proapoptotic genes and promoting viral yield. US3 also counteracts apoptosis driven by the Bcl‐2 family protein BAD. While early studies suggested that US3 phosphorylates BAD at canonical Akt/PKA sites (Ser112, Ser136, Ser155), later evidence showed that US3 can prevent apoptosis even when these residues are mutated (e.g., BAD3S/A), indicating a noncanonical or downstream mechanism independent of direct BAD phosphorylation at these sites [77]. One possibility is that US3 inhibits caspases that cleave BAD or modulates other mitochondrial apoptotic regulators. This highlights that while US3 *functionally* achieves the same outcome as Akt (inactivating BAD), the molecular mechanism may differ from direct phosphorylation of the canonical sites. By targeting multiple key host proteins that are typically regulated by Akt, US3 effectively serves as a potent surrogate kinase, reinforcing host cell survival and ensuring an environment conducive to prolonged viral replication and particle production.

### 1.13 US3 Modulation of PKA Signaling

The optimal phosphorylation motif recognized by US3 (Arg‐Arg‐X‐Ser/Thr) closely resembles that of cAMP‐dependent protein kinase A (PKA). Indeed, US3 has been shown to phosphorylate traditional PKA substrates in infected cells [26]. Moreover, PKA activation itself can suppress apoptosis and rescue viral replication defects in cells infected with US3‐deficient HSV‐1 mutants, suggesting a significant functional overlap between these kinases, at least in the context of antiapoptosis [15, 17]. It is even plausible that US3 may indirectly activate PKA or its downstream effectors, although this remains to be fully elucidated. These findings imply that US3 may hijack or partially mimic elements of both Akt and PKA signaling pathways to exert robust antiapoptotic effects and manipulate cellular metabolism—highlighting its versatility and integration into multiple host signaling pathways.

In summary, US3 functions in an Akt‐like manner by phosphorylating FOXO1/FOXO3, TSC2, BAD, and other Akt‐pathway components, thereby activating mTORC1 and suppressing apoptosis. These Akt‐like functions and survival‐related substrates are summarized in Table 1.

### 1.14 Modulation of Mitotic/Apoptotic Machinery for Nuclear Egress

**Figure 3:**
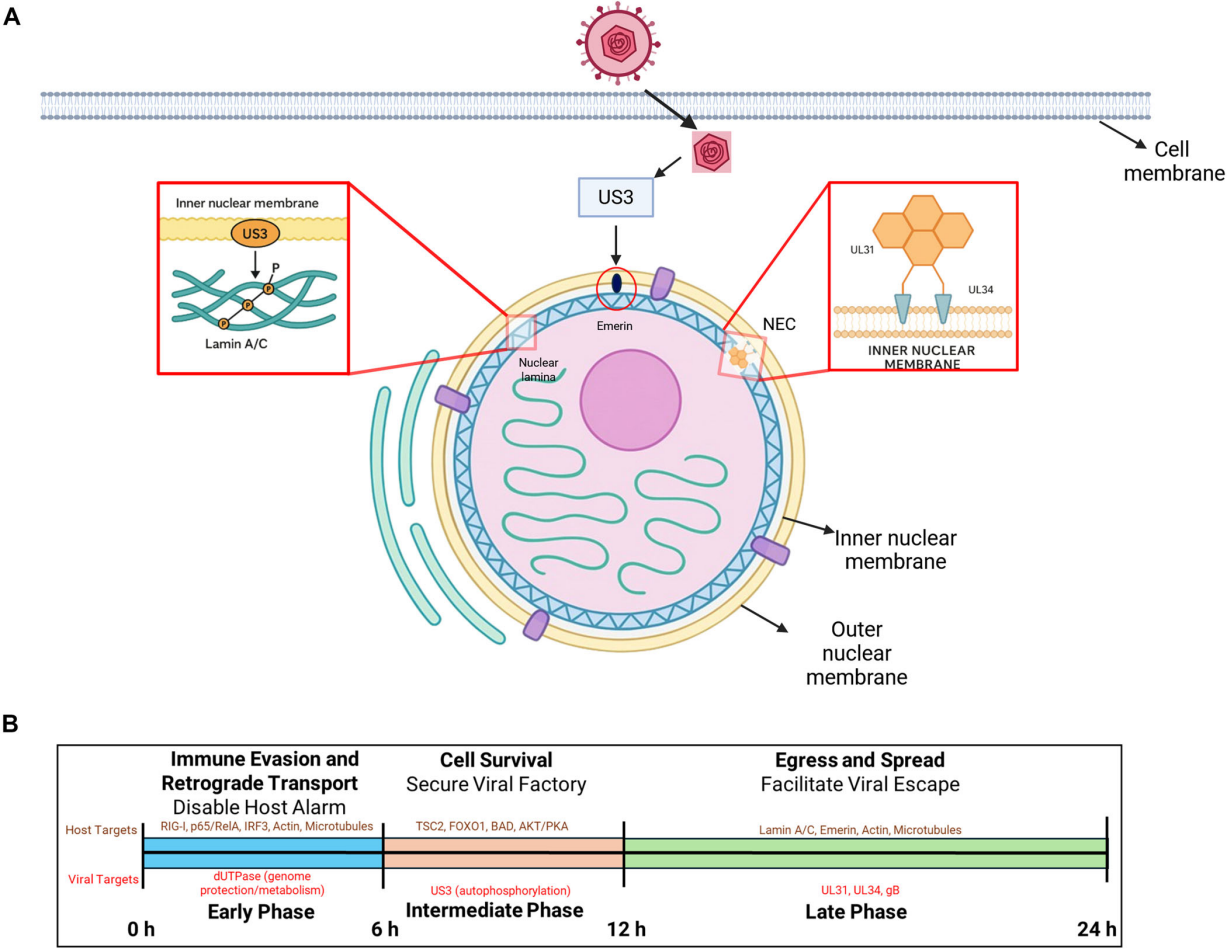
US3‐mediated nuclear egress and temporal framework of HSV‐1 infection. (a) US3 facilitates HSV‐1 nuclear egress by targeting the nuclear lamina and the nuclear egress complex (NEC). During infection, US3 phosphorylates Lamin A/C and Emerin, two major components of the inner nuclear membrane–lamina interface, to promote local lamina disassembly and facilitate capsid passage through the inner nuclear membrane. US3 also phosphorylates NEC components UL31 and UL34, regulating their localization and function at the nuclear rim. These coordinated modifications ensure efficient capsid budding into the perinuclear space and subsequent cytoplasmic release. (b) Conceptual temporal framework of US3 activities during HSV‐1 infection, showing its modulation of host and viral targets to optimize infection. In the Early Phase (0–6 h), US3 ensures a favorable environment by phosphorylating host antiviral defenses like RIG‐I, p65/RelA, and IRF3, while also modulating Actin/Microtubules for retrograde transport. Crucially, in this phase, US3 also targets the viral enzyme vdUTPase to regulate its activity, thereby ensuring viral genome protection and metabolic efficiency. Moving into the Intermediate Phase (6–12 h), US3 promotes cell survival by mimicking Akt/PKA‐like activity to phosphorylate host factors TSC2, FOXO1, and BAD, and regulates its own function through autophosphorylation. Finally, in the Late Phase (12–24 h), US3 facilitates egress and spread by disrupting the nuclear envelope via phosphorylation of host proteins Lamin A/C and Emerin. It also targets the viral Nuclear Egress Complex components UL31 and UL34, as well as the envelope protein gB, to mediate efficient release and spread of progeny virions. (Except where otherwise indicated (Figure 1a, SWISS‐MODEL), all figures are original conceptual schematics and analyzes generated by the authors for this review).

HSV‐1 replicates its genome and assembles nucleocapsids within the host cell nucleus. To continue viral assembly and initiate egress, these large capsids must traverse the nuclear envelope—a formidable barrier reinforced by the nuclear lamina [79]. Herpesviruses overcome this obstacle through a unique two‐step nuclear egress process: (1) primary envelopment at the inner nuclear membrane (INM) and (2) de‐envelopment (membrane fusion) at the outer nuclear membrane (ONM) into the cytoplasm. This intricate pathway requires controlled remodeling and disassembly of the nuclear lamina [79, 80]. HSV‐1 US3 kinase plays a critical role in this process, likely by emulating host kinases that regulate lamina disassembly during mitosis or apoptosis (Figure 3a).

### 1.15 US3 Phosphorylation of Nuclear Lamins

The nuclear lamina, primarily composed of lamin A/C and lamin B filaments, provides essential structural support to the nucleus and anchorage for chromatin and nuclear pore complexes [81]. During mitosis, cellular kinases such as cyclin‐dependent kinase 1 (Cdk1, also known as Cdc2) phosphorylate lamins at specific sites, triggering lamina disassembly and nuclear envelope breakdown [82]. Similarly, protein kinase C delta (PKCδ) phosphorylates lamins during apoptosis [83]. HSV‐1 infection induces a more localized and potentially reversible lamina remodeling necessary for capsid budding, rather than full nuclear envelope breakdown. US3 is directly implicated in this controlled disruption. It phosphorylates lamin A/C at multiple serine residues, both in vitro and in infected cells, and this modification is dependent on US3 kinase activity. Phosphorylation leads to partial solubilization and altered localization of lamin A/C at the nuclear periphery during infection [6]. While lamin A/C contains only one canonical US3 consensus motif, US3 phosphorylates it promiscuously at additional sites [6], demonstrating its ability to modify targets without strict adherence to a defined motif. This suggests US3 functionally substitutes for or complements mitotic/apoptotic kinases such as Cdk1 or PKCδ to locally disrupt lamina structure. Moreover, US3 may indirectly regulate PKCδ activity at the nuclear periphery, further enhancing lamin reorganization [84]. US3 also phosphorylates Emerin, another key inner nuclear membrane protein that interacts with lamins [45]. Phosphorylation by US3 alters Emerin's localization, potentially contributing to the rearrangement of the nuclear envelope structure required for egress.

### 1.16 US3 Coordination With the Nuclear Egress Complex (NEC)

Nuclear egress is orchestrated by the conserved viral nuclear egress complex (NEC), composed of the HSV‐1 proteins pUL31 and pUL34. The NEC assembles into a hexagonal scaffold at the INM, deforming the membrane to facilitate capsid budding into the perinuclear space [85]. Both pUL31 and pUL34 are phosphorylated by US3. Specifically, pUL31 phosphorylation by US3 is crucial for its proper localization at the nuclear rim, which is essential for efficient NEC function [12]. US3 phosphorylation of UL34 also plays a role, potentially tempering UL34‐mediated disruption of the nuclear envelope [12]. In the absence of US3 kinase activity, NEC components mislocalize, and while primary envelopment at the INM may still occur, the resulting enveloped virions accumulate abnormally in large clusters within the perinuclear space, failing to efficiently de‐envelop at the ONM and release into the cytoplasm [12, 86]. This severe egress defect underscores the indispensable role of US3‐mediated phosphorylation of NEC components and/or nuclear lamins for both efficient budding and downstream release from the perinuclear compartment. Although US3 kinase activity is not essential for viral replication in standard cultured cells, US3‐deficient HSV‐1 mutants exhibit markedly reduced replication and neuroinvasion in vivo. In cell culture, these mutants can reach titers similar to wild‐type virus, suggesting that US3 is dispensable for basal replication. However, in mouse models, both US3‐null viruses and recombinant strains carrying non‐phosphorylatable mutations in key US3 targets—such as Lamin A/C or UL31–UL34—show severe growth impairment. These findings highlight that while US3 is non‐essential for replication in vitro, its kinase activity and substrate phosphorylation are critical for efficient neuronal spread and pathogenesis in vivo [87].

Another viral factor that contributes to nuclear egress is the HSV‐1 γ34.5 (ICP34.5) protein, which promotes lamin disassembly by recruiting protein kinase C (PKC) to the nuclear envelope. While the HSV‐1 γ34.5 (ICP34.5) protein also promotes egress—partly by enhancing PKC‐mediated lamin phosphorylation—US3 uniquely targets both lamins and NEC components, underscoring its central and indispensable role in coordinating egress [84]. The viral kinase UL13 also interacts with UL31 and UL34 and is involved in egress [4, 86], and UL13 phosphorylates US3 at Ser147, enhancing its activity [48]. While UL13 contributes to egress, the specific defects observed in US3 mutants highlight a distinct, essential role for US3 kinase activity in coordinating NEC function and lamina modification, possibly under the regulatory influence of UL13.

Comparative studies across herpesviruses reveal conserved roles for CHPKs (e.g., HCMV UL97, EBV BGLF4, VZV ORF47) and NEC homologs (UL31/UL34 orthologs) in nuclear egress, often involving lamin phosphorylation. In parallel, US3 and its functional homologs, such as VZV ORF66 and PRV US3, also contribute to nuclear lamina disassembly, underscoring both conserved and virus‐specific adaptations in herpesviral nuclear exit.

Overall, US3 facilitates nuclear egress through phosphorylation of lamin A/C, emerin, the UL31/UL34 nuclear egress complex, and MATR3, leading to lamina disassembly and nuclear envelope remodeling. PRV US3 and VZV ORF66 both phosphorylate nuclear lamins and MATR3, highlighting conservation of lamina remodeling across alphaherpesviruses. These nuclear substrates are summarized in Table 1.

### 1.17 US3‐Mediated Regulation of Neuronal Retrograde Transport

HSV‐1 capsid enters the neuron at the axon terminal and travels to neuronal cell body along microtubules by retrograde trafficking [88]. This journey is essential for the virus to establish a latent infection within the nervous system. The viral kinase US3 has been identified as a critical regulator of this process. HSV‐1 and PRV mutants that lack a functional US3 protein exhibit a significant defect in retrograde transport, a primary factor contributing to their reduced neuroinvasion and attenuation in vivo [89, 90]. The latency‐associated transcript (LAT) expressed during latency is also critical for long‐term neuronal persistence by blocking apoptosis, although no direct mechanistic links to US3 or UL13 have been demonstrated.

The exact molecular mechanism is still an area of active research. The reasons for this defect likely involve US3's kinase activity. This activity appears to affect the interaction between the incoming viral capsid and the host cellular transport machinery, particularly the dynein motor complex that drives retrograde movement along microtubules. Cellular kinase GSK3β phosphorylates the cellular dynein intermediate chain at S87/T88 (IC‐1B) and S88/T89. These sites are in the N‐terminal domain of the Intermediate Chain, which overlaps with the Ndel1‐binding region. Phosphorylation at these residues reduces Ndel1 binding, thereby altering dynein motility [91]. It is thought that US3 could phosphorylate cellular GSK3β to regulate dynein motor protein indirectly [7]. HSV‐1 relies on the cytoplasmic dynein motor for retrograde axonal transport to neuronal cell bodies [92, 93, 94]. HSV‐1 US3 is essential for efficient neuroinvasion, as viruses lacking US3 or carrying mutations in its kinase domain exhibit replication and retrograde transport defects in neurons and trigeminal ganglia [47, 89]. Therefore, it is possible that US3 directly regulates the dynein motor protein. One possibility is that US3 phosphorylates viral proteins involved in dynein binding, such as UL36 (VP1/2) and UL37 tegument proteins [95]. Another possibility is that US3 modulates host dynein or dynactin complexes and their adapter proteins. Phosphorylation of one or more host components within these complexes can alter their activity, localization, or assembly, thereby facilitating the movement of viral particles. Finally, US3 may influence the microtubular network itself. Its known interactions with the cytoskeleton, though mostly studied during egress could also affect microtubule dynamics in axons, making them more permissive for retrograde transport. By modulating signaling pathways such as Akt/mTOR, ERK, and PKA that regulate cytoskeletal organization, US3 may further enhance viral transport efficiency.

In summary, US3 regulates cytoskeletal transport and remodeling by modulating dynein adapters, disrupting F‐actin, and directly phosphorylating kinesins such as KIF3A, thereby enhancing viral trafficking. PRV US3 has also been implicated in capsid transport, suggesting conserved roles in microtubule‐dependent trafficking. These transport‐related substrates are compiled in Table 1.

### 1.18 Actin Remodeling and Viral Transport

Beyond its effects on microtubule‐based transport, HSV‐1 US3 plays a pivotal role in reorganizing the actin cytoskeleton to facilitate viral trafficking. Early studies by the Favoreel group demonstrated that US3 expression induces extensive actin rearrangements, including the breakdown of stress fibers and the formation of membrane projections, thereby enhancing intercellular spread of the virus [96, 97]. These alterations in actin architecture are mediated, at least in part, through phosphorylation‐dependent modulation of actin regulatory proteins such as cofilin and signaling components of the RhoA–ROCK–LIMK axis [98].

Disassembly of cortical actin filaments is particularly important during the early stages of infection, when capsids entering at the cell periphery must reach microtubules for retrograde transport. In this context, US3‐mediated actin remodeling removes a physical barrier to capsid trafficking and promotes efficient delivery of viral particles to the nucleus. In parallel, US3's influence on actin structures supports the formation of membrane protrusions that facilitate direct cell‐to‐cell spread, a process critical for immune evasion and persistence in neuronal tissues [99]. These findings underscore US3's dual role in cytoskeletal regulation: dismantling actin barriers to promote microtubule‐dependent retrograde transport, and simultaneously reshaping actin networks to optimize viral dissemination. Similar actin‐related effects have also been observed for PRV US3, suggesting that actin remodeling is a conserved feature of alphaherpesvirus US3 kinases [100].

### 1.19 Summary of HSV‐1 US3 Kinase Substrates and Functions

To provide an integrated overview of the HSV‐1 US3 kinase functions, we compiled a summary of all experimentally reported host and viral substrates (Table 1). This table brings together evidence from diverse studies, outlining the species or cellular context, type of experimental validation, known phosphorylation sites, and the resulting biological consequences. By consolidating these findings, the table complements the preceding sections and highlights how US3 targets multiple functional modules, including immune signaling, chromatin regulation, nuclear egress, and cytoskeletal transport.

### 1.20 Concluding Remarks and Future Perspectives

HSV‐1 US3 is expressed with an early late kinetic profile, enabling its kinase activity to function throughout infection. As a tegument protein, US3 is packaged into virions and becomes active immediately upon entry, where it suppresses innate immune responses and cytoskeletal defenses. During later stages of infection, newly synthesized US3 contributes to nuclear egress, transcriptional modulation, and intracellular transport. This temporal regulation (Figure 3b) positions US3 to influence multiple phases of the viral life cycle, from early immune evasion to late virion assembly and spread.

US3 viral kinase employs multiple mechanisms to manipulate host signaling networks, ensuring optimal viral replication and immune suppression. It acts as a surrogate for cellular kinases such as Akt and PKA and directly targets regulators including NF‐κB p65, IRF3, RIG‐I, β‐catenin, TSC2, FOXO1, and nuclear lamins. Through these activities, US3 reprograms host signaling to promote viral replication, persistence, and immune evasion. Its ability to phosphorylate a wide array of viral and cellular substrates underscores its critical role in orchestrating infection.

Several important questions remain unresolved. The full spectrum of US3 substrates and their temporal regulation during infection requires systematic investigation, ideally through unbiased quantitative phosphoproteomics. The mechanistic consequences of phosphorylation—how individual modifications alter substrate function, localization, or binding interactions—are not yet fully defined. High‐resolution structural studies of US3, particularly in complex with substrates such as TSC2, RIG‐I, lamin A/C, or NEC proteins, are needed to clarify substrate recognition, promiscuity, and non‐consensus targeting. Further, the mechanisms that regulate US3 itself, including its interplay with the viral UL13 kinase and host phosphatases, remain incompletely understood and needs further detailed research efforts.

From a translational perspective, US3 is an appealing antiviral target. However, several obstacles complicate drug development. The high conservation of kinase active sites makes the selective inhibition of viral kinases without causing host toxicity a challenging task. The diverse functions of US3 also indicate the possibility of compensatory mechanisms if a single function is blocked. These limitations underscore the need for structure‐guided approaches and high‐throughput screening platforms to identify inhibitors with both specificity and safety.

Conflicting reports in the literature further highlight areas that require clarification. US3 has been described as both suppressing and sustaining ERK1/2 activation depending on cellular context, and the mechanisms underlying MHC‐I downregulation remain inconsistent across studies. Addressing such discrepancies through standardized experimental systems will be important to refine our understanding of US3 biology.

Finally, the relative contribution of US3's diverse functions—including antiapoptosis, immune evasion, and nuclear egress—to in vivo pathogenesis and viral spread remains unclear. Although deletion of US3 has little effect on viral replication in cultured cells, it profoundly reduces neuroinvasion and replication in mice, underscoring its critical role during in vivo infection. Comparative studies across alphaherpesviruses further reveal that while homologous kinases such as PRV US3 and VZV ORF66 share overlapping functions in cytoskeletal modulation and nuclear lamina remodeling, several features of HSV‐1 US3 appear to be nonconserved or remain insufficiently characterized in current literature. Genetically modified animal models and temporal knockout approaches will be essential to dissect these functions. Advancing mechanistic and structural understanding of US3 will not only clarify its diverse “moonlighting” roles during infection but also inform strategies to exploit this kinase as a potential therapeutic target.

## Author Contributions

Md Imran Hossain conceived and designed the article, performed the literature review, and drafted the article. Christella J. Nelson, Ojasvi Dutta, Harikrishnan Mohan, and Md Mehedi Hasan contributed to critical review and editing. Konstantin G. Kousoulas contributed to article design, supervision, critical revisions, and secured funding support. All authors approved the final version of the article.

## Conflicts of Interest

The authors declare that they have no conflicts of interest.

## Data Availability

The data that support the findings of this study are available on request from the corresponding author. The data are not publicly available due to privacy or ethical restrictions.
